# Comparative analysis of the clinical aspects of colorectal cancer in young adult and older adult patients in Saudi Arabia

**DOI:** 10.3389/fonc.2024.1460636

**Published:** 2024-12-05

**Authors:** Marwh G. Aldriwesh, Amer R. Aljaian, Khalid M. Alorf, Mohammed A. Bayounis, Yazeed H. Alrayani, Winnie Philip, Mohammed Algarni, Nahar A. Alselaim, Raniah S. Alotibi

**Affiliations:** ^1^ Department of Clinical Laboratory Sciences, College of Applied Medical Sciences, King Saud bin Abdulaziz University for Health Sciences, Riyadh, Saudi Arabia; ^2^ King Abdullah International Medical Research Center, Riyadh, Saudi Arabia; ^3^ College of Medicine, King Saud bin Abdulaziz University for Health Sciences, Riyadh, Saudi Arabia; ^4^ Research and Innovation Unit, College of Applied Medical Sciences, King Saud bin Abdulaziz University for Health Sciences, Riyadh, Saudi Arabia; ^5^ Department of Oncology, Ministry of the National Guard–Health Affairs, Riyadh, Saudi Arabia; ^6^ Department of Medicine, College of Medicine, King Saud bin Abdulaziz University for Health Sciences, Riyadh, Saudi Arabia; ^7^ Department of General Surgery, Ministry of the National Guard–Health Affairs, Riyadh, Saudi Arabia

**Keywords:** colorectal cancer, gastrointestinal cancer, young adult, older adult, early-onset, late-onset, Saudi Arabia

## Abstract

**Introduction:**

Recent studies have shown an increase in the prevalence of early-onset colorectal cancer (CRC) in people aged 20–49 compared to those aged 50–74, with a more rapid increase in the younger age groups. Poorly differentiated, left-sided, and rectal tumors were more common in young adults than in older adult CRC patients. We aimed to improve the understanding of early-onset CRC and to guide primary care physicians on strategies to mitigate its impact.

**Methods:**

Adult patients with CRC identified within 2015–2022 were recruited and divided into young adult-onset (CRC identified at age ≤49 years) and older adult-onset (CRC identified at age ≥50 years). Clinical data were retrieved from electronic medical records, then analyzed. Multivariable analyses were performed to predict the CRC prognosis in both age groups.

**Results:**

The study cohort had 530 patients categorized into young adult (n=98; 18.5%) and older adult (n=432; 81.5%). Higher proportions of family histories of CRC, other malignancies, and inflammatory bowel disease in the young adult group were observed (P<0.05). Gastrointestinal symptoms mainly abdominal pain and nausea were more often identified in the young adults. Mucinous adenocarcinoma, signet ring cells, and poorly differentiated tumors were higher in the young adults (P<0.05). Lymphovascular invasion was an independent predictor for advanced stage CRC (AOR 8.638, 95%CI 2.152–34.673, P=0.002 for young adults and AOR 21.757, 95%CI 10.025–47.219, P=0.001 for older adults). Further, the mucinous (AOR 3.727, 95%CI 1.937–7.173, P=0.001 for young adults and AOR 3.534, 95%CI 1.698–7.354, P=0.001 for older adults) and lymphovascular invasion (AOR 3.371, 95%CI 2.107–5.393, P=0.001 for young adults and AOR 3.246, 95%CI 1.910–5.517, P=0.001 for older adults) were independent predictors for recurrence/late metastasis in both age groups.

**Conclusion:**

We recommended to raise awareness among healthcare providers of the importance of lowering the threshold of suspicion in young people presenting with worrisome gastrointestinal symptoms. Our findings suggested the importance of reconsidering the current CRC screening guidelines to lower the threshold of the recommended starting age.

## 1 Introduction

Colorectal cancer (CRC) is the second leading cause of cancer-associated mortality worldwide (1). In developed nations, the incidence rate of CRC in individuals over the age of 50 has been declining since the mid-1980s, possibly due to the introduction of screening tests (such as fecal occult blood tests and colonoscopies) that enable the detection and removal of precancerous lesions as well as an increased awareness of risk factors associated with CRC in the community (1–3). However, recent global studies have shown an increase in the prevalence of early-onset CRC from 4.2/100,000 to 6.7/100,000 in individuals aged 20–49 compared to those aged 50–74 between 1990 and 2019, with a more rapid increase in the younger age groups (4). Furthermore, the mortality rate associated with early-onset CRC showed an upward trend worldwide. A total of 223,000 young adults were diagnosed with CRC in 2019, resulting in 86,000 deaths and a loss of 4.2 million disability-adjusted life years (4). The uptick in early-onset CRC prevalence was consistent across all five sociodemographic index (SDI) regions and 190 of 204 countries and territories. Middle and high-middle SDI regions exhibited a faster annual increase in early-onset CRC, which warrants further investigation (4). In the cancer incidence report for Saudi Arabia, CRC ranks first among males and the third among females, with 1,729 cases reported in 2020—representing 12.3% of all newly diagnosed cancer cases (5). The gender distribution reveals a prevalence among males, with 966 cases (55.9%) being males, resulting in a male-to-female ratio of 126:100 and age-standardized incidence rates of 12.4 per 100,000 for males and 9.6 per 100,000 for females (5). The report puts the median age at CRC diagnosis as 60 years old for males and 58 years old for females, with an age range of 17–98 for males and 20–98 for females (5). Available data in the literature indicated that CRC was more advanced (stage III or IV) in 60.0% of young adult patients (<50 years) than in older adult patients (46.0%–50.0%) (3). In addition, poorly differentiated, left-sided, and rectal tumors were more common in young adults than in older adult CRC patients (3). In all CRC patients, the signet ring cell was seen in <1.0%. However, in the young adult subgroup, the signet ring cell was detected in 3.0%–13.0% of cases (3).

Modifiable and nonmodifiable risk factors for CRC have been highlighted in the literature (3, 4, 6–10). Patients with inflammatory bowel disease (IBD) or abdominal irritation have been reported to be more susceptible to developing CRC than those without (3, 4, 6–10). In contrast, diabetes mellitus (DM)/insulin resistance (IR), obesity, Western diet, high consumption of red and processed meat, low dietary fiber intake, low physical activity, and smoking have been identified as modifiable risk factors for CRC development (3, 10–15). For early-onset CRC in particular, hereditary cancer predisposition syndromes, lifestyle factors, and the composition and function of the gut microbiome have recently emerged as risk factors (6, 7, 10, 16).

The root causes of the increasing incidence of CRC in young adults remain uncertain. There is a lack of established diagnostic and therapeutic procedures specifically tailored for early-onset CRC in the young adult population, which is currently an unmet need in clinical practice. Furthermore, there is no consensus regarding the differentiation of early-onset CRC as either indistinguishable or a separate clinical entity from CRC in older patients. In this article, we conducted comparative analysis of clinical characteristics of early-onset CRC and late-onset CRC in a cohort of Saudi Arabian patients. We aimed to improve the understanding of early-onset CRC and to guide primary care physicians on strategies to mitigate its impact.

## 2 Materials and methods

### 2.1 Study design, settings, and participants

The present study is a retrospective observational cohort study that was performed at King Abdulaziz Medical City (KAMC), King Abdullah International Medical Research Centre (KAIMRC), and King Saud bin Abdulaziz University for Health Sciences (KSAU–HS), which are part of the Ministry of National Guard–Health Affairs (MNGHA), Riyadh, Saudi Arabia. The available electronic medical records of patients receiving a confirmed diagnosis of CRC between 1^st^ of January 2015 and 31^st^ of December 2022 were accessed. The inclusion criteria were adult patients (age >14 years) with CRC identified within the study period (01 January 2015–31 December 2022). Patients with CRC were divided into two groups according to age at CRC diagnosis: young adult-onset (CRC identified at age ≤49 years) and older adult-onset (CRC identified at age ≥50 years). The age groups were determined according to the literature (17) and the categorization of CRC patients at KAMC, where the young adult CRC patient population comprises individuals diagnosed prior to CRC screening at age 50 years.

### 2.2 Study variables

Data were retrieved from patients’ electronic medical records through the BestCare system and included admission, follow-up, pathology, surgical and emergency notes. Patients’ demographics, family histories, comorbidities, presenting symptoms at CRC diagnosis, computed tomography (CT) scan findings, and history of colonoscopies were collected. Body mass index (BMI) was calculated and categorized into the following categories: underweight <18.0 kg/m^2^, normal weight 18.0–24.9 kg/m^2^, overweight 25.0–29.9 kg/m^2^, and obese ≥30.0 kg/m^2^. The presence of malignancies other than CRC in the patient’s family, such as breast cancer, hepatocellular carcinoma, lymphoma, and renal cell carcinoma, was also recorded.

CRC-associated diagnostic data, including primary tumor side and location, CRC stage, tumor size, and histopathology results, were collected. The patients included in the study were all diagnosed with CRC and classified according to the International Classification of Diseases 10^th^ Revision (ICD-10). Identification of the CRC stage was made by histopathological examination of a lesion obtained through biopsy during a colonoscopy, with further histopathological features identified subsequent to tumor resection. This was followed by imaging to identify whether the tumor was a metastasis. For stage IV CRC, metastatic lesions were biopsied to identify the source of the lesions. Mucinous and signet ring adenocarcinomas were determined in CRC patients by biopsy and histopathology examinations. The degree of tumor differentiation was identified by pathologists as grade 1 (well differentiated: >95.0% gland formation), grade 2 (moderately differentiated: 50.0% to 95.0% gland formation), grade 3 (poorly differentiated: <50.0% gland formation), and grade 4 (undifferentiated: no gland or mucin formation and no squamous or neuroendocrine differentiation). Lymphovascular invasion was also recorded as either present (pathologists were able to identify the invasion of the tumor into vessels), not present (pathologists were unable to identify the invasion of the tumor into vessels), or not definitive (the invasion remained a query, as the pathologists could not be sure whether invasion was present or not). Patients in the earlier CRC stages were likely to benefit from surgery, as their tumors had not yet spread widely. Surgery was also performed if there were complications arising from the tumor, such as obstruction, regardless of the stage, to relieve and treat the patient. Surgery complications, such as surgical site infections, leaks from the site of an anastomosis, and bleeding from the site of surgery, were also considered.

### 2.3 Statistical analyses

The collected data were entered into Microsoft^®^ Excel for Mac version 16.80 following an ethically approved data collection form. Then, the collected data were exported to the IBM^®^ SPSS^®^ statistics software version 21 (SPSS Inc., Chicago, Illinois, USA) for statistical analyses. To address the study objectives, the patients were divided into two groups: young adult-onset (CRC identified at age ≤49 years) and older adult-onset (CRC identified at age ≥50 years). The descriptive statistics involved presenting the categorical variables as frequencies and percentages, while the numerical variables with non-normal data distribution were presented as medians with an interquartile range (IQR). Inferential statistics were conducted to examine the association of study variables between the two age groups using a non-parametric test (the Mann–Whitney U test) for the numerical variables and the Pearson chi-square or Fisher’s exact tests for the categorical variables. Statistical significance was defined as a P-value of <0.05.

Bivariable and multivariable analyses were conducted using the binary logistic model to predict the risk factors for advanced stage CRC, recurrence and late metastasis, or mortality in both young adult-onset and older adult-onset CRC patients. The dependent variables were stage III and stage IV CRC, recurrence and late metastasis, and mortality, while the independent variables tested were male gender, BMI, left-sided tumor, tumor in the sigmoid and rectosigmoid, mucinous, presence of signet ring, lymphovascular invasion, family history of CRC or other malignancy, smoking, DM, dyslipidemia, and IBD. Only variables that show significant P-value (<0.05) in the bivariable analysis were considered in the multivariable analysis. Multivariable analyses were adjusted for the following variables: male gender, BMI, left-sided tumor, tumor location, mucinous, presence of signet ring, lymphovascular invasion, family history of CRC/other malignancy, smoking, DM, dyslipidemia, and IBD. A P-value <0.05 in the multivariable analysis indicated a statistically significant and independent risk factor for stage III and stage IV CRC, recurrence and late metastasis, or mortality in the corresponding age group.

## 3 Results

### 3.1 Demographics, family history, and comorbidities of young adult-onset versus older adult-onset CRC

The study cohort consisted of 530 patients with CRC, who were categorized into young adult-onset (diagnosed at age ≤49 years, n=98; 18.5%) and older adult-onset (diagnosed at age ≥50 years, n=432; 81.5%). Gender distribution revealed a higher proportion of males in total, with 49.0% males in the young adult-onset group compared to 61.3% in the older adult-onset group (**Table 1**). The median age at CRC diagnosis for young adult-onset was 42 years (IQR 35, 45), with the youngest age being 18 years, while the median age for older adult-onset was 65 years (IQR 58, 74), with the eldest being 107 years. BMI showed no significant difference between the two groups (P=0.660), with similar proportions of CRC patients categorized as obese (35.9% in the young adult-onset group versus 34.3% in the older adult-onset group). The analysis of the patients’ family history and comorbidities indicated a significantly higher proportion of family histories of CRC, other malignancies, and IBD in the young adult-onset group than in the older adult-onset group at P=0.007, P=0.019, and P=0.001, respectively. However, DM/IR, hypertension, dyslipidemia, and cardiovascular diseases were significantly more frequent in the older adult-onset group at, P=0.001, as detailed in **Table 1**.

**Table 1:** Demographics and clinical characteristics of young adult-onset versus older adult-onset colorectal cancer (CRC) patients, N=530.

	Young adult CRC patients ≤49 years, n=98 (18.5%)	Older adult CRC patients ≥50 years, n=432 (81.5%)	P-value
**Gender, n (%)**			**0.025**
Male	48 (49.0)	265 (61.3)	
Female	50 (51.0)	167 (38.7)	
**Age at diagnosis (years), median (IQR^1^)**	42 (35, 45)	65 (58, 74)	**0.001**
**Body Mass Index, n (%)**			0.660
Underweight	4 (4.3)	10 (2.4)	
Normal weight	29 (31.5)	130 (31.4)	
Overweight	26 (28.3)	132 (31.9)	
Obese	33 (35.9)	142 (34.3)	
Family history and comorbidities, n (%)			
Family history of CRC	7 (8.0)	7 (1.8)	**0.007**
Family history of malignancy	8 (8.2)	12 (2.8)	**0.019**
Smoking	6 (6.1)	34 (7.9)	0.554
Diabetes mellitus/insulin resistance	14 (14.3)	251 (58.1)	**0.001**
Inflammatory bowel disease (IBD)	6 (6.1)	1 (0.2)	**0.001**
Hypertension	18 (18.4)	269 (62.3)	**0.001**
Dyslipidemia	5 (5.1)	149 (34.5)	**0.001**
Cardiovascular disease	1 (1.0)	69 (16.0%)	**0.001**
Thyroid dysfunction	4 (4.1)	43 (9.9)	0.164
**Post IBD tumor screening, n (%)**	3 (3.1)	1 (0.2)	**0.021**
Symptoms at diagnosis, n (%)			
Gastrointestinal			
Abdominal pain	63 (64.3)	227 (52.5)	**0.035**
Weight loss	29 (29.6)	108 (25)	0.349
Loss of appetite	14 (14.3)	70 (16.2)	0.639
Vomiting	21 (21.4)	76 (17.6)	0.375
Hematemesis	0 (0.0)	5 (1.2)	0.590
Nausea	17 (17.3)	38 (8.8)	**0.012**
Constipation	31 (31.6)	155 (35.9)	0.426
Diarrhea	14 (14.3)	37 (8.6)	0.083
Rectal bleeding	41 (41.8)	181 (41.9)	0.991
Abdominal mass	18 (18.4)	54 (12.5)	0.126
Perineal pain	1 (1.0)	2 (0.5)	0.459
Change in bowel habit	8 (8.2)	26 (6.0)	0.434
Intestinal obstruction	3 (3.1)	18 (4.2)	0.779
Systemic			
Anemia	2 (2.0)	40 (9.3)	**0.017**
Hematuria	0 (0.0)	1 (0.2)	0.815
Night sweats	0 (0.0)	4 (0.9)	0.440
Fever	6 (6.1)	19 (4.4)	0.435
Fatigue	0 (0.0)	23 (5.3)	**0.012**
Dizziness	3 (3.1)	12 (2.8)	0.746
Respiratory			
Cough	0 (0.0)	4 (0.9)	0.440
Chest pain	0 (0.0)	2 (0.5)	0.664
Shortness of breath	0 (0.0)	11 (2.5)	0.230
**Abnormal computed tomography scan, n (%)**	42 (42.9)	224 (51.9)	0.108
**Colonoscopy performed, n (%)**	64 (65.3)	286 (66.2)	0.865
**Previous colonoscopy performed, n (%)**	10 (10.2)	43 (10)	0.941
**Primary tumor side, n (%)**			0.394
Right	15 (15.8)	99 (23.1)	
Left	78 (82.1)	320 (74.8)	
Right and left	0 (0.0)	1 (0.2)	
Transverse colon	2 (2.1)	8 (1.9)	
Primary tumor location, n (%)			
Cecum	6 (6.7)	44 (10.4)	0.196
Ascending colon	0 (0.0)	24 (5.7)	**0.013**
Descending colon	7 (7.8)	30 (7.1)	0.996
Sigmoid	26 (29.21)	113 (26.84)	0.950
Rectum	17 (19.1)	75 (17.8)	0.913
Rectosigmoid	22 (24.72)	71 (16.86)	0.206
Colorectal cancer stage, n (%)			
I	4 (4.4)	38 (9.3)	0.131
II	23 (25.2)	108 (26.3)	0.844
III	37 (40.6)	158 (38.4)	0.695
IV	27 (29.6)	107 (26.0)	0.478
**Primary tumor size (cm), median (IQR)**	5 (3, 6)	4 (3, 6)	0.891
Histology, n (%)			
Adenocarcinoma	92 (93.9)	378 (87.5)	0.072
Mucinous	19 (19.6)	45 (10.4)	**0.012**
Presence of signet ring	5 (5.2)	6 (1.4)	**0.034**
**Degree of tumor differentiation, n (%)**			**0.018**
Well differentiated	0 (0.0)	3 (0.7)	
Moderately differentiated	76 (86.4)	379 (94.0)	
Poorly differentiated	12 (13.6)	21 (5.2)	
**Lymphovascular invasion, n (%)**			0.150
Present	34 (42.0)	118 (32.3)	
Not present	37 (45.7)	210 (57.5)	
Not definitive	10 (12.3)	37 (10.1)	
Metastasis, n (%)			
Liver	22 (22.4)	90 (20.8)	0.724
Lung	13 (13.3)	44 (10.2)	0.378
Ovary	8 (8.2)	6 (1.4)	**0.001**
Peritoneum	9 (9.2)	22 (5.1)	0.119
Bone	2 (2.0)	10 (2.3)	0.612
Spine	0 (0.0)	3 (0.7)	0.541
Mesentery	2 (2.0)	8 (1.9)	0.579
Distant lymph nodes	5 (5.1)	11 (2.6)	0.192
Omentum	1 (1.0)	1 (0.2)	0.336
Diaphragm	0 (0.0)	1 (0.2)	0.815
Bladder	1 (1.0)	1 (0.2)	0.336
Pancreas	0 (0.0)	2 (0.5)	0.664
Adrenal gland	2 (2.0)	1 (0.2)	0.089
**Surgery, n (%)**	67 (68.4)	305 (70.8)	0.639
**Surgery complications, n (%)**	0 (0.0)	17 (3.9)	0.052
**Chemotherapy, n (%)**	79 (80.0)	234 (54.3)	**0.001**
**Immunotherapy, n (%)**	10 (10.2)	20 (4.6)	**0.032**
**Radiotherapy, n (%)**	22 (22.4)	65 (15.1)	0.076
**Palliative care, n (%)**	13 (13.3)	74 (17.2)	0.347
**Mortality, n (%)**	24 (24.5)	99 (22.9)	0.739

^1^IQR, Interquartile range. Bold P-values indicate statistical significance (<0.05).

### 3.2 Clinical and diagnostic characteristics of young adult-onset versus older adult-onset CRC

The differences in the clinical presentation of CRC between the young adult-onset and the older adult-onset group are highlighted in **Table 1**. Abdominal pain was the most frequently reported gastrointestinal symptom in both age groups, but it was significantly more often identified in the young adult-onset group (64.3%) compared to the older adult-onset group (52.5%) (P=0.035). Nausea was also detected more often in the young adult-onset group (17.3%) than in the older adult-onset group (8.8%) (P=0.012). However, anemia and fatigue were more frequently recorded at CRC presentation by the older adult-onset group than by the young adult-onset group, at P=0.017 and P=0.012, respectively. The two age groups showed little difference in the primary tumor’s side, with the majority of tumors located on the left side of the colon (82.1% in the young adult-onset group and 74.8% in the older adult-onset group) (P=0.394). Specific tumor location analysis revealed that the sigmoid was the most common site in both age groups, accounting for 53.93% in the young adult-onset group and 43.7% in the older adult-onset group, followed by the rectum, while tumors in the ascending colon were detected in the older adult-onset group only (5.7%).

The highest percentage of patients in both age groups was diagnosed at advanced stages of CRC (70.2% of the young adult-onset group and 64.4% of the older adult-onset group). The presence of mucinous adenocarcinoma and signet ring cells were significantly higher in the young adult-onset group compared to the older adult-onset group with P=0.012 and P=0.034, respectively. The majority of CRC patients in both age groups presented with moderately differentiated tumors, at 86.4% and 94.0% for the young adult-onset and older adult-onset groups, respectively. However, the young adult-onset CRC group had a higher proportion of poorly differentiated tumors (13.6%) compared to the older adult-onset group (5.2%), at P=0.018. Lymphovascular invasion was present in 42.0% of the young adult-onset group and in 32.3% of the older adult-onset group, although this difference did not reach significance level (P=0.150).

### 3.3 Management and outcomes of young adult-onset versus older adult-onset CRC

Tumor resection was performed for the majority of patients in both age groups (68.4% for young adult-onset and 70.8% for older adult-onset), as outlined in **Table 1**. The young adult-onset CRC group was significantly more exposed to chemotherapy and immunotherapy than the older adult-onset group, at P=0.001 and P=0.32, respectively. Both age groups had records of metastasis in a variety of organs, with the liver being the most common organ for metastasis (22.4% in the young adult-onset group versus 20.8% in the older adult-onset group), followed by lungs (13.3% in the young adult-onset group versus 10.2% in the older adult-onset group). However, ovarian metastasis was significantly more often detected in females with CRC whose age was ≤49 years (8.2%) than in females ≥50 years old (1.4%), at P=0.001.

### 3.4 Predictors of advanced stage CRC in young adult-onset patients versus older adult-onset patients

The associations between different independent factors and advanced stage CRC (stages III and IV) in both age groups were examined (**Table 2**). The bivariable analyses revealed that lymphovascular invasion was statistically associated with advanced stage CRC in both age groups (OR 9.444, 95%CI 2.426–36.775, P=0.001 for young adult-onset and OR 21.119, 95%CI 9.769–45.657, P=0.001 for older adult-onset). This association remained significant in both age groups in the multivariable analyses, which indicated that lymphovascular invasion might be an independent predictor for advanced stage CRC (AOR 8.638, 95%CI 2.152–34.673, P=0.002 for young adult-onset and AOR 21.757, 95%CI 10.025–47.219, P=0.001 for older adult-onset). Furthermore, multivariable analyses indicated that smoking could be an independent risk factor for advanced tumor stages only in the older adult-onset CRC group (AOR 3.642, 95%CI 1.225–10.828, P=0.020).

**Table 2:** Predictors of advanced stage (stage III and stage IV) colorectal cancer (CRC) in young adult-onset versus older adult-onset patients.

	Young adult CRC patients ≤49 years			Older adult CRC patients ≥50 years		
Bivariable analysis	OR	95%CI	P-value	OR	95%CI	P-value
Male vs female	0.402	0.159–1.017	0.054	0.901	0.595–1.365	0.622
BMI (underweight vs others)	1.015	0.935–1.102	0.715	1.013	0.985–1.042	0.360
Left-sided tumor vs right-sided tumor	0.843	0.242–2.939	0.789	1.131	0.699–1.832	0.615
Tumor location (sigmoid and rectosigmoid vs others)	0.736	0.286–1.892	0.524	0.737	0.487–1.114	0.147
Mucinous (yes vs no)	1.797	0.536–6.024	0.342	1.595	0.797–3.194	0.187
Presence of signet ring (yes vs no)	1.763	0.188–16.547	0.620	2.799	0.324–24.191	0.350
Lymphovascular invasion (yes vs no)	9.444	2.426–36.775	**0.001**	21.119	9.769–45.657	**0.001**
Family history of CRC (yes vs no)	2.690	0.308–23.485	0.371	0.826	0.229–2.974	0.769
Family history of malignancy (yes vs no)	2.690	0.308–23.485	0.371	0.450	0.135–1.502	0.194
Smoking (yes vs no)	1.733	0.185–16.267	0.630	2.658	1.071–6.597	**0.035**
Diabetes (yes vs no)	0.821	0.225–2.998	0.766	0.738	0.487–1.119	0.153
Dyslipidemia (yes vs no)	0.615	0.097–3.905	0.606	0.804	0.528–1.224	0.308
IBD (yes vs no)	2.203	0.245–19.807	0.481	Not applicable^1^		
**Multivariable analysis^2^**	**AOR**	**95%CI**	**P-value**	**AOR**	**95%CI**	**P-value**
Male						
BMI, underweight						
Left-sided tumor						
Tumor location, sigmoid and rectosigmoid						
Mucinous						
Presence of signet ring						
Lymphovascular invasion	8.638	2.152–34.673	**0.002**	21.757	10.025–47.219	**0.001**
Family history of CRC						
Family history of malignancy						
Smoking				3.642	1.225–10.828	**0.020**
Diabetes						
Dyslipidemia						
IBD						

^1^The bivariable analysis was not performed for older adult-onset CRC due to a low number of old adults who had IBD (n=1). ^2^Multivariable analyses were adjusted for the following variables: male, BMI, left-sided tumor, tumor in the sigmoid and rectosigmoid, mucinous, presence of signet ring, lymphovascular invasion, family history of CRC/other malignancy, smoking, diabetes, dyslipidemia, and IBD. CRC, Colorectal cancer; OR, Odds ratio; AOR, Adjusted odds ratio; CI, Confidence interval; BMI, Body mass index; IBD, Inflammatory bowel disease. Bold P-values indicate statistical significance (<0.05).

### 3.5 Predictors of recurrence and late metastasis in young adult-onset versus older adult-onset CRC patients

The outcomes of bivariable analyses indicated a significant association between the presence of mucinous (OR 4.093, 95%CI 1.398–11.979, P=0.010 for young adult-onset and OR 3.503, 95%CI 1.860–6.597, P=0.001 for older adult-onset) or lymphovascular invasion (OR 3.354, 95%CI 2.126–5.290, P=0.001 for young adult-onset and OR 3.194, 95%CI 1.903–5.364, P=0.001 for older adult-onset patients) and recurrence/late metastasis in CRC patients in both age groups. These associations also showed significant levels in the multivariable analyses for both groups, as detailed in **Table 3**. Hence, the mucinous (AOR 3.727, 95%CI 1.937–7.173, P=0.001 for young adult-onset and AOR 3.534, 95%CI 1.698–7.354, P=0.001 for older adult-onset patients) and lymphovascular invasion (AOR 3.371, 95%CI 2.107–5.393, P=0.001 for young adult-onset and AOR 3.246, 95%CI 1.910–5.517, P=0.001 for older adult-onset patients) are independent risk factors for recurrence and late metastasis in CRC patients of both age groups (**Table 3**).

**Table 3:** Predictors of recurrence/late metastasis in young adult-onset versus older adult-onset CRC patients.

	Young adult CRC patients ≤49 years			Older adult CRC patients ≥50 years		
Bivariable analysis	OR	95%CI	P-value	OR	95%CI	P-value
Male vs female	0.542	0.239–1.227	0.141	0.645	0.414–1.007	0.054
BMI (underweight vs others)	0.993	0.947–1.042	0.789	1.008	0.988–1.028	0.445
Left-sided tumor vs right-sided tumor	1.829	0.601–5.561	0.288	0.859	0.513–1.441	0.566
Tumor location (sigmoid and rectosigmoid vs others)	1.213	0.521–2.826	0.654	0.708	0.448–1.120	0.140
Mucinous (yes vs no)	4.093	1.398–11.979	**0.010**	3.503	1.860–6.597	**0.001**
Presence of signet ring (yes vs no)	6.222	0.668–57.921	0.108	0.619	0.072–5.361	0.663
Lymphovascular invasion (yes vs no)	3.354	2.126–5.290	**0.001**	3.194	1.903–5.364	**0.001**
Family history of CRC (yes vs no)	1.468	0.533–4.044	0.458	2.119	0.586–7.657	0.252
Family history of malignancy (yes vs no)	2.250	0.912–5.548	0.078	2.28	0.710–7.360	0.166
Smoking (yes vs no)	1.866	0.961–3.623	0.066	2.059	0.992–4.272	0.052
Diabetes (yes vs no)	0.945	0.645–1.384	0.770	1.169	0.746–1.831	0.496
Dyslipidemia (yes vs no)	0.864	0.564–1.324	0.502	0.988	0.622–1.570	0.959
IBD (yes vs no)	6.839	1.312–35.655	**0.022**	Not applicable^1^		
**Multivariable analysis^2^**	**AOR**	**95%CI**	**P-value**	**AOR**	**95%CI**	**P-value**
Male						
BMI, underweight						
Left-sided tumor						
Tumor location, sigmoid and rectosigmoid						
Mucinous	3.727	1.937–7.173	**0.001**	3.534	1.698–7.354	**0.001**
Presence of signet ring						
Lymphovascular invasion	3.371	2.107–5.393	**0.001**	3.246	1.910–5.517	**0.001**
Family history of CRC						
Family history of malignancy						
Smoking						
Diabetes						
Dyslipidemia						
IBD	2.545	0.370–17.488	0.342			

^1^The bivariable analysis was not performed for older adult**-**onset CRC due to a low number of old adults who had IBD (n=1). ^2^Multivariable analyses were adjusted for the following variables: male, BMI, left-sided tumor, tumor in the sigmoid and rectosigmoid, mucinous, presence of signet ring, lymphovascular invasion, family history of CRC/other malignancy, smoking, diabetes, dyslipidemia, and IBD. CRC, Colorectal cancer; OR, Odds ratio; AOR, Adjusted odds ratio; CI, Confidence interval; BMI, Body mass index; IBD, Inflammatory bowel disease. Bold P-values indicate statistical significance (<0.05).

### 3.6 Predictors of mortality in young adult-onset versus older adult-onset CRC patients

The mortality rate was similar for the two age groups, with 24.5% for the young adult-onset group and 22.9% for the older adult-onset group. The tested independent variables showed insignificant associations with mortality in the young adult CRC patients, as detailed in **Table 4**. However, upon bivariable analyses of the older adult-onset group, lymphovascular invasion (OR 3.494, 95%CI 1.992–6.128, P=0.001) and smoking (OR 2.951, 95%CI 1.439–6.054, P=0.003) were significantly associated with mortality. These findings suggest that lymphovascular invasion (AOR 3.614, 95%CI 2.018–6.472, P=0.001) and smoking (AOR 4.083, 95%CI 1.686–9.884, P=0.002) are independent predictors of mortality in the older adult CRC patients.

**Table 4:** Predictors of mortality in young adult-onset versus older adult-onset CRC patients.

	Young adult CRC patients ≤49 years			Older adult CRC patients ≥50 years		
Bivariable analysis	OR	95%CI	P-value	OR	95%CI	P-value
Male vs female	0.846	0.336–2.130	0.723	0.732	0.465–1.154	0.179
BMI (underweight vs others)	1.005	0.956–1.058	0.834	0.997	0.972–1.022	0.787
Left-sided tumor vs right-sided tumor	0.418	0.130-1.339	0.142	0.925	0.541–1.582	0.776
Tumor location (sigmoid and rectosigmoid vs others)	0.462	0.174–1.224	0.120	0.756	0.473–1.209	0.243
Mucinous (yes vs no)	2.093	0.714–6.139	0.178	1.605	0.817–3.153	0.170
Presence of signet ring (yes vs no)	5.071	0.794–32.391	0.086	1.696	0.306–9.399	0.545
Lymphovascular invasion (yes vs no)	1.659	0.472–35.831	0.430	3.494	1.992–6.128	**0.001**
Family history of CRC (yes vs no)	1.255	0.227–6.927	0.795	0.367	0.046–2.935	0.345
Family history of malignancy (yes vs no)	1.971	0.434–8.946	0.379	3.516	1.108–11.158	**0.033**
Smoking (yes vs no)	1.591	0.273–9.281	0.606	2.951	1.439–6.054	**0.003**
Diabetes (yes vs no)	0.818	0.208–3.216	0.774	2.951	1.439–6.054	0.084
Dyslipidemia (yes vs no)	0.761	0.081–7.157	0.811	1.178	0.739–1.877	0.492
IBD^1^ (yes vs no)	1.591	0.273–9.281	0.606	Not applicable^1^		
**Multivariable analysis^2^**	**AOR**	**95%CI**	**P-value**	**AOR**	**95%CI**	**P-value**
Male						
BMI, underweight						
Left-sided tumor						
Tumor location, sigmoid and rectosigmoid						
Mucinous						
Presence of signet ring						
Lymphovascular invasion				3.614	2.018–6.472	**0.001**
Family history of CRC						
Family history of malignancy				2.932	0.687–12.501	0.146
Smoking				4.083	1.686–9.884	**0.002**
Diabetes						
Dyslipidemia						
IBD						

^1^The bivariable analysis was not performed for older adult**-**onset CRC due to a low number of old adults who had IBD (n=1). ^2^Multivariable analyses were adjusted for the following variables: male, BMI, left-sided tumor, tumor in the sigmoid and rectosigmoid, mucinous, presence of signet ring, lymphovascular invasion, family history of CRC/other malignancy, smoking, diabetes, dyslipidemia, and IBD. CRC, Colorectal cancer; OR, Odds ratio; AOR, Adjusted odds ratio; CI, Confidence interval; BMI, Body mass index; IBD, Inflammatory bowel disease. Bold P-values indicate statistical significance (<0.05).

## 4 Discussion

The main objective of this study was to examine the clinical differences between young adult-onset and older adult-onset CRC patients. The results showed that the young adult-onset group exhibited distinct clinical features compared with the older adult-onset group. Genetic predisposition was more frequently reported in the young adults. Furthermore, young adults presented with more acute symptoms at CRC diagnosis, whereas older adults were more likely to present with chronic conditions. CRC-associated pathologic characteristics, including mucinous, presence of signet ring, and poorly differentiated tumors, were more commonly observed in the young adults. Young adults were also more exposed to invasive cancer therapeutic modalities, including chemotherapy and immunotherapy. However, the left-sided tumor, specifically in the sigmoid part of the colon, was the most common tumor location in both age groups (**Table 1**).

The data presented here revealed a higher frequency of CRC cases in males, especially in the older adult-onset group, which is consistent with previous studies that reported a male predominance in CRC incidence across different age groups (18). In addition, the study indicated a significant proportion of family histories of CRC and other malignancies in the young adult-onset group compared to the older adult-onset group, indicating a key role of genetic predisposition in early-onset CRC. This aligns with previous studies on the familial clustering of the disease (4, 6, 8, 10, 16, 19). The findings also supported existing evidence linking IBD to early-onset CRC, as indicated by the increased prevalence of IBD in younger patients compared to their older counterparts (20). On the other hand, DM/IR, hypertension, dyslipidemia, and cardiovascular disease were more frequent in the older adult-onset group (**Table 1**). The higher incidence of DM/IR in older adults is consistent with the literature associating DM/IR with an increased CRC risk in older populations (13, 21).

The differences in the clinical presentation of CRC between the young adult-onset group and the older adult-onset group provide important insights into the symptomatology and disease progression between the two age groups. Abdominal pain and nausea were more commonly reported by young adults, which is consistent with the literature and suggests that young CRC patients often present with more acute symptoms at diagnosis (3, 14). However, older adults in our study were more likely to present with chronic conditions, including anemia and fatigue, consistent with findings from previous studies (2, 7). Almost similar rates of constipation, weight loss, and rectal bleeding were found between the two age groups in the present study, reflecting the commonality of these symptoms in CRC patients across different ages (7, 22–24). A previous study conducted in Indonesia on a sample of 170 patients to compare early- and late-onset CRC found that anemia and tumors in the ascending and descending colons were more prevalent in the early-onset CRC group (25). This conflicts with our findings that anemia and ascending colon involvement are significantly more common in older adult CRC patients than in young adult CRC patients.

Previous studies have reported variations in the histopathologic features of CRC between young adult-onset and older adult-onset CRC patients (10, 26, 27). Tumors in young adults expressed more hostile histologic characteristics compared to older adults, which included mucinous, signet cells, and poorly differentiated tumors (10, 28, 29). These findings are consistent with the results of this study, as shown in **Table 1**. Also, Gao et al. concur with our findings regarding the presentation of abdominal pain and the presence of poorly differentiated tumors and mucinous or signet ring cancer cells, which are significantly more prevalent in young adult CRC patients than in older adult CRC patients (30). In this context, CRC in young adults may have more aggressive behavior, different responses to cancer therapies, and different survival rates when compared to CRC in older adults (10). Although the higher rate of metastatic presentation in young adults observed in this study was not statistically significant, it supports the literature suggesting that young patients may have more advanced disease at diagnosis (3, 6, 10, 14, 31). The predominance of liver metastasis in both age groups underscores the importance of liver surveillance in CRC management, consistent with established patterns of metastatic spread in CRC (23). Furthermore, the significant difference in ovarian metastasis rates detected, with a higher incidence in young females (**Table 1**), highlights the need for vigilance in monitoring atypical metastatic sites in these patients, an area less frequently discussed in the current literature (32).

A review published by Saraiva and colleagues in 2023 highlighted the lack of specific evidence-based therapeutic protocols for early-onset CRC (10). However, the literature indicates that different therapeutic approaches are used to manage CRC, depending on the age of the patient (33). For instance, young adult CRC patients have more opportunities for surgical intervention (either for early-stage CRC or metastatic conditions), radiotherapy, and intensive adjuvant therapy options, such as multiagent chemotherapy (10, 33, 34). Our findings further support these studies, as chemotherapy, immunotherapy, and radiotherapy were more commonly provided to young adults compared to older adults, while the latter group received more palliative care. From a clinical point of view, young adults may have a lower incidence of comorbidities, a higher functional status, and fewer adverse effects of systemic therapy, resulting in better tolerability of multiagent protocols compared to older individuals (10).

In addition to our primary goal, we also investigated the associations between a variety of potential risk factors and advanced stage CRC, CRC recurrence and late metastasis, and mortality in both young adult-onset and older adult-onset patients. In multivariable analyses, lymphovascular invasion was found to be a significant independent risk factor for advanced stage CRC and cancer recurrence and late metastasis in both age groups (**Tables 2**, **3**). An Italian-based study on 2,073 CRC patients reports similar results to those of our study and concluded that lymphovascular invasion was an independent risk factor for advanced stage CRC and recurrence (35). In addition to lymphovascular invasion, the presence of mucinous differentiation in CRC was also identified as an independent risk factor for cancer recurrence and late metastasis in both age groups (**Table 3**). However, lymphovascular invasion was identified as a significant independent risk factor for mortality only in the older adult-onset group (**Table 4**). It has been demonstrated in the literature that lymphovascular invasion plays a key role in predicting patient survival outcomes (36, 37). In other words, lymphovascular invasion is strongly associated with decreased rates of disease-free survival and overall survival in CRC patients (36, 37). In addition, previous evidence has highlighted the prognostic value of lymphovascular invasion and mucinous differentiation in increasing the likelihood of metastasis and tumor dissemination (38). From a diagnostic perspective, lymphovascular invasion and mucinous differentiation in biopsy specimens could guide treatment and management decisions and assess cancer prognosis (36, 37).

The findings of this study have significant implications for public health interventions aimed at lowering the incidence of CRC among young individuals in particular. Given the remarkable incidence of early-onset CRC reported in our findings, it is essential that targeted preventive and screening strategies be developed for young individuals. Prompt recognition of risk factors such as IBD, a family history of CRC, and specific lifestyle habits is critical. Public health initiatives should concentrate on raising awareness about the growing risk of CRC in young individuals and educating them on the significance of identifying gastrointestinal symptoms that may be indications of an underlying medical condition. Diet plays a major role in CRC prevention; therefore, young people should be encouraged to follow a diet rich in fiber, fruit, and vegetables, in addition to reducing their processed meat intake and consumption of high-fat foods, as doing so would exponentially lower their risk tendency. Considering modifiable risk factors such as smoking, obesity, and a sedentary lifestyle is also crucial. Campaigns designed to encourage physical activity and healthy weight management can be fundamental to mitigating such risk factors. Collaborative efforts involving schools and community centers are another avenue to provide educational programs on proper nutrition and healthy lifestyle habits that may promote healthy living among young people. This combined strategy encompassing dietary education, lifestyle modification, and raising awareness can boost prevention efforts and, eventually, minimize the burden of CRC among young adults.

In Saudi Arabia, CRC screening is recommended for individuals aged 45 to 75 years (39). This is based on the Saudi Ministry of Health, which advises annual immunological stool testing for individuals in this age group (39). If the test result is negative, the test is repeated annually, and if positive, the individual is referred for a colonoscopy (39). This recommendation targets individuals who are at moderate risk of CRC, who typically fall within the 45–75 age bracket (39). Previous national guidelines for CRC screening in Saudi Arabia were published in 2015 and recommended that CRC screening should probably be started at the age of 45 (40). In contrast to the Saudi national guidelines, the U.S. Preventive Services Task Force recommends CRC screening for all adults aged 50 to 75, with a focus on annual or biannual screenings, depending on the test type (41). The American Cancer Society, however, advocates starting CRC screening at age 45 for individuals at average risk and continuing until age 75, after which screening should depend on personal health factors (42). In our study, we found that 98 out of 530 CRC patients were diagnosed with CRC at age ≤49 (**Table 1**). In light of these findings, our study underscores the importance of updating the current CRC screening guidelines to lower the recommended starting age to the early 40s. Our study also recommends raising awareness among healthcare providers regarding the importance of lowering the threshold for suspicion of CRC in young people presenting with worrisome gastrointestinal symptoms, which would facilitate a more proactive approach to diagnosis.

The Materials and Methods section (Sub-section 2.1) clarifies how the patients who participated in this study were selected. However, there is the possibility of selection biases stemming from several factors. For instance, our dependence on electronic medical records may have incidentally excluded individuals who did not seek medical care and patients diagnosed outside the institution that served as the research site, which may have resulted in the underrepresentation of certain demographic elements and clinical presentations. Furthermore, given the retrospective nature of this study, there may be other biases pertaining to the completeness of the medical records, which may limit the reliability of the data collected with regard to symptomatology, comorbidities, and family history. These probable biases need to be considered when interpreting the study findings and their implications to facilitate a balanced understanding of the clinical factors that impact CRC in the two age groups studied. In addition, focusing on a single geographic region of Saudi Arabia may limit the generalizability of the findings, as variations in awareness, healthcare accessibility, and screening programs may impact the characteristics and incidence of CRC in different geographic areas in the country. However, it is important to note that the medical center utilized as the research site is one of the largest tertiary hospitals in Saudi Arabia, and it receives patients from various regions across the nation.

This study does not directly consider confounding factors, such as lifestyle, socioeconomic status, and access to healthcare, because data on these factors were not present in the patients’ medical records. However, the potential impact of such factors is implied in this study by capturing the differences in clinical features and family history between early-onset and late-onset CRC. We found that young individuals tend to present with very acute symptoms and exhibit high rates of genetic predispositions, which may be linked to their lifestyle habits and accessibility to healthcare. Although factors such as obesity, diet, and exercise are known as modifiable risk factors, the analysis performed in this study was predominantly focused on clinical findings rather than directly investigating how socioeconomic status and access to healthcare influence the diagnosis and treatment of CRC. Therefore, there is a need for future studies that incorporate these confounding factors to provide a more comprehensive view of their roles in CRC incidence and outcomes in young versus older adult populations.

To the best of our knowledge, our study is the first research conducted in Saudi Arabia to focus on comparing the clinical aspects of CRC in young adults vis-à-vis the typical older adult population. Recently published studies in Saudi Arabia primarily address the epidemiology and trends in the incidence of young-onset CRC (43–45). However, none of these studies (43–45) have thoroughly examined the similarities and differences in disease presentation between young-onset and late-onset cases. In this paper, we present the results of our comparative analysis of these two patient populations. Considering the rising incidence of young-onset CRC, which is expected to place a considerable burden on the Saudi healthcare system, this study holds significant relevance. With a large segment of the Saudi population under the age of 30, our findings underscore the importance of addressing this concern.

In conclusion, the findings of this study enhance the understanding of early-onset CRC by highlighting distinct clinical characteristics and predictors observed in young adult vis-à-vis older adult CRC patients. Young adults were more frequently observed to have genetic predispositions and presented with acute symptoms at diagnosis, indicating a need for heightened awareness among healthcare providers. The histopathologic features of CRC, including mucinous tumors, poorly differentiated tumors, and the presence of signet ring cells, were significantly more common in young adult CRC patients than in older adult CRC patients. In addition, young adults often require invasive treatment modalities such as chemotherapy and immunotherapy. Notably, left-sided tumors, especially in the sigmoid colon, were prevalent across both age groups. These findings underscore the importance of lowering the threshold for suspicion of CRC in young individuals presenting with worrisome gastrointestinal symptoms. Therefore, we recommend reconsidering the current CRC screening guidelines to lower the starting age, thus facilitating earlier diagnosis and intervention for this emerging patient population. The study compared early-onset and late-onset CRC in a cohort of Saudi Arabian patients, filling a gap in the literature, especially given the paucity of available data on this topic in Saudi Arabia. The findings of our study may contribute to the global understanding of CRC onset according to different age groups, thereby enriching the existing body of knowledge in this area of research.

## Data Availability

The original contributions presented in the study are included in the article. Further inquiries can be directed to the corresponding author.
